# Therapeutic Status and Available Strategies in Pancreatic Ductal Adenocarcinoma

**DOI:** 10.3390/biomedicines9020178

**Published:** 2021-02-11

**Authors:** Gitika Thakur, Raj Kumar, Saet-Byul Kim, Sang-Yeob Lee, Sung-Lim Lee, Gyu-Jin Rho

**Affiliations:** 1 Department of Theriogenology and Biotechnology, College of Veterinary Medicine and Research Institute of Life Science, Gyeongsang National University, Jinju 52828, Korea; gitika18oct@gnu.ac.kr (G.T.); sbkim4@gnu.ac.kr (S.-B.K.); sylee2@gnu.ac.kr (S.-Y.L.); sllee@gnu.ac.kr (S.-L.L.); 2 Department of Biotechnology and Bioinformatics, Jaypee University of Information Technology, Waknaghat, Solan 173 234, Himachal Pradesh, India; raj.kumar@juit.ac.in

**Keywords:** pancreatic cancer, pancreatic ductal adenocarcinoma, stem cells, pancreatic cancer stem cells

## Abstract

One of the most severe and devastating cancer is pancreatic cancer. Pancreatic ductal adenocarcinoma (PDAC) is one of the major pancreatic exocrine cancer with a poor prognosis and growing prevalence. It is the most deadly disease, with an overall five-year survival rate of 6% to 10%. According to various reports, it has been demonstrated that pancreatic cancer stem cells (PCSCs) are the main factor responsible for the tumor development, proliferation, resistance to anti-cancer drugs, and recurrence of tumors after surgery. PCSCs have encouraged new therapeutic methods to be explored that can specifically target cancer cells. Furthermore, stem cells, especially mesenchymal stem cells (MSCs), are known as influential anti-cancer agents as they function through anti-inflammatory, paracrine, cytokines, and chemokine′s action. The properties of MSCs, such as migration to the site of infection and host immune cell activation by its secretome, seem to control the microenvironment of the pancreatic tumor. MSCs secretome exhibits similar therapeutic advantages as a conventional cell-based therapy. Moreover, the potential for drug delivery could be enhanced by engineered MSCs to increase drug bioactivity and absorption at the tumor site. In this review, we have discussed available therapeutic strategies, treatment hurdles, and the role of different factors such as PCSCs, cysteine, GPCR, PKM2, signaling pathways, immunotherapy, and NK-based therapy in pancreatic cancer.

## 1. Introduction

The pancreas is an organ located in the abdomen having both exocrine and endocrine functions. It plays an essential role in the digestion of food by releasing enzymes from its exocrine part, maintains blood glucose level by producing two major hormones: glucagon and insulin secreted from the endocrinal region of the pancreas. Normal healthy cells become cancerous when a series of changes take place in the DNA sequence, leads the cell to divide uncontrollably and migrate to adjacent cells. Cancer is the second leading cause of death worldwide and was accountable for an estimated 9.6 million deaths in 2018 (World Health Organization (WHO), 2018). It is the major public health issue and the main cause of death in Korea [1], second leading in the United States [2], and one of the leading causes of death in India [3]. One of the leading causes of cancer mortality and the most deadly malignant neoplasm is pancreatic cancer [4]. In 2012, around 338,000 individuals had pancreatic cancer worldwide, making it the eleventh most prevalent cancer. Around 458,918 new pancreatic cancer cases were identified worldwide in 2018, representing 2.5% of all cancers [5]. The American Cancer Society estimated about 57,600 new cases (30,400 male and 27,200 female) of pancreatic cancer and predicted that 47,050 patients (24,640 male and 22,410 female) will die of pancreatic cancer in 2020 [2]. In 2019, pancreatic cancer was the fourth leading cause of cancer deaths. It has been projected to become the second leading cause by 2030 [6,7].

Pancreatic cancer arises when cells in the pancreas start to divide uncontrollably and form a mass. There are different types of cancer cells based on their origin, for example, carcinoma (cancer of epithelial cells), sarcoma (cancer of mesenchymal cells in blood vessels, muscles, and other tissues), myeloma/leukemia/lymphoma (blood cell-related cancer), and adenocarcinoma (cancer of mucus-producing glandular cells). Two main subtypes of pancreatic cancer have been narrowly classified into exocrine and endocrine. Pancreatic ductal adenocarcinoma (PDAC) is an exocrine cell tumor mainly of the ductal cells, more common (>85%) than endocrine cell tumors (<5%) [8]. About 50% of PDACs are detected when the tumor is locally invasive or metastatic. PDAC has a 5-year survival rate of 6% (ranges from 2% to 10%) [6,9]. Exocrine cancer is the most common form of pancreatic cancer, which comprises 95% of all pancreatic cancers [10,11]. Out of all exocrine cancers, the most common and aggressive form is ductal cancer, i.e., PDAC. It is one of the most malignant tumors, characterized by uncontrollable growth [9]. Approximately 85% to 90% of pancreatic cancers are PDAC [11]. Recently, researchers have reviewed the current therapeutic options, dysregulated pathways, tumor microenvironment, and many other factors associated with PDAC [6,12]. Approximately 60% to 70% of cases emerge from the head of the pancreas, which comprises the bile duct; these cases are typically diagnosed earlier than body and tail tumors [13]. Tail and body tumors are linked with a poorer prognosis [14]. In patients with PDAC, the most common symptoms are abdominal pain, weight loss, and jaundice [15], whereas the new onset of type 2 diabetes is a less common symptom [16]. 

Additionally, studies have shown that PDAC and diabetes are co-related; at the time of cancer diagnosis, one- to two-thirds of patients with PDAC are diabetic [17]. The key concern is whether the growth of cancer is susceptible to diabetes or the consequence of the tumor is diabetes. The five leading behavioral and dietary risks, such as high body mass index, low consumption of fruit and vegetables, physical inactivity, alcohol, and tobacco, are responsible for about one-third of cancer deaths [4]. About 8% of pancreatic cancers occur in families who carry mutations in tumor suppressor genes, including *P16Ink4a/CDKN2A*, *BRCA2*, *MLH1*, *MSH2*, *STK1*, or *VHL* [18]. In 95% of PDAC cases, activating mutations in the KRAS oncogene are detected, but agents that can successfully target this high prevalence change in PDAC are not yet available. Available traditional strategies: surgery, radiation, and chemotherapy have been widely used, but no significant improvements have been shown. Overall survival remains poor for metastatic cancer, with less than 20% of patients surviving after the end of the first year [19]. For the better treatment of PDAC, alternative treatment approaches are desperately needed. Furthermore, stem cell therapy, which has shown therapeutic efficacy for solid tumors (breast, prostate, and lung carcinomas), can be one of the best options to treat PDAC [20]. This review will assist researchers to better understand the available treatment strategies, treatment hurdles, and the role of stem cells, mainly MSCs (Mesenchymal stem cells), in pancreatic cancer, especially in PDAC. Stem cells can be used for regenerative medicine, cancer stem-cell-targeted treatment, anticancer drug screening applications, and immunotherapy.

## 2. Treatment Hurdles

Treatment with cytotoxic agents: FOLFIRINOX (a mixture of Leucovorin and other chemotherapy medicines: Fluorouracil (5FU), Irinotecan and Oxaliplatin]) or Gemcitabine/Nab-paclitaxel is the current drug therapy for PDAC. In recent decades, these cytotoxic agents and other approved drugs (e.g., Erlotinib) used to treat PDAC have been shown to improve survival by a few months [21]. Furthermore, late diagnosis is responsible for a poor prognosis of PDAC. Due to the prevalence of metastatic spread and the local involvement of major blood vessels, over 80% of cases are not suitable for surgical resection of tumors [22]. In order to identify the specific characteristics of patients with less than 5 years of survival in the past 30 years, a Finnish study analyzed PDAC patient records. More than 50% of the cases with 5-year survival were incorrectly diagnosed with PDAC; even for those with the correct diagnosis, only one person with PDAC survived to 11 years [23]. Therefore, discovering new treatments for PDAC is a major unmet medical need.

## 3. Stem Cells

**Figure 1:**
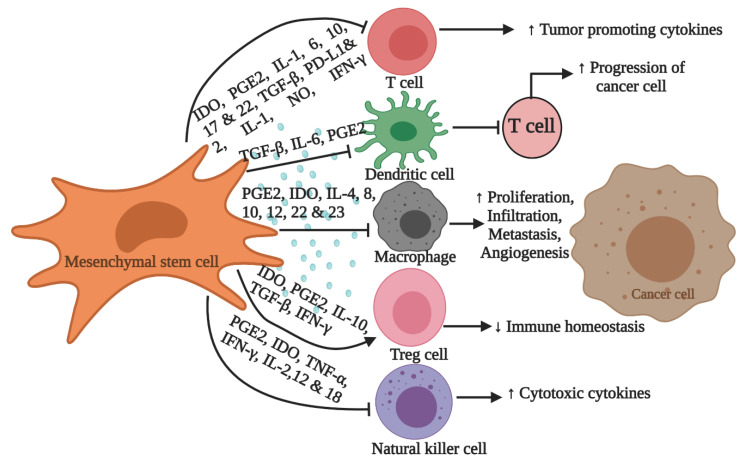
Immune cells in mesenchymal stem cells (MSCs) and cancer cell microenvironment. MSCs regulate the immune system by inducing Treg cells along with suppressing cytotoxic and helper T cells through TGFβ, IL-10, PGE2, NO, and IDO. Cytokines secretions are also regulated by MSCs, by inhibiting macrophage and natural killer cells via PGE2 and IDO. In cancer cells, tumor growth and progression are increased through the inhibition of T cells by dendritic cells, and this action of dendritic cells is suppressed by immunomodulatory factors secreted by MSCs. IL: interleukin; TGF-β: transforming growth factor beta; PGE2: prostaglandin E2; NO: nitric oxide; PD-L: programmed death-ligand; IDO: indoleamine 2,3-dioxygenase; Treg: T regulatory cell; →: activation; ⊣: inhibition. Figure created with BioRender.com and modified from [24].

There are various stem cell therapies based on natural killer cells, activated T cells, and dendritic cells, which are extremely effective in treating cancer. Stem cells can be isolated from the embryonic (Embryonic stem cells: ESCs) and adult (Mesenchymal stem cells: MSCs) tissues, but their properties are different. Stem cells are known as influential anti-cancer agents as they function through anti-inflammatory, paracrine, cytokines, and chemokine′s action and are proficient in regulating the tumor microenvironment. Stem cells have shown tremendous promise as therapeutic options for the next generation. In 2019, Chopra et al. reviewed the stem cell-based clinical trials, where different types of stem cells are used for the treatment of various cancers [24]. Around 544 clinical trials are currently enlisting patients (above 500 for hematopoietic stem cells and 12 for MSCs) for stem cell therapy to cure various cancers. Outcome measures, improved overall survival period, the accomplishment of complete or partial cancer-free status, and minimized serious negative effects have been evaluated in these studies. Until now, few studies have been performed on pancreatic cancer (particularly for PDAC) based on stem cell therapy. Merely four experiments using hematopoietic stem cells have been registered on clinicaltrials.gov, while none were registered with MSCs [24]. MSCs have unique immunomodulatory, inflammatory properties, homing capacity, and migration capability; consequently, they can migrate to the site of infection or inflammation [25,26]. Immunomodulatory factors, iNOS, IDO, TGF-β, LIF, PGE-2, and many others are secreted by MSCs to inhibit T cell proliferation [27,28]. Such factors can modulate the microenvironment of the tumor cells (Figure 1). In 2009, Cousin et al. investigated the capability of human adipose derived MSCs to cure PDAC in mice models and a cell line. They suggested that MSCs induce cell cycle inhibition at the G1 stage and downregulation of *cyclin D1* and *CDK4* that lead to cell death [29]. Furthermore, umbilical cord MSCs ability to reduce murine pancreatic cancer cell growth was tested using a mouse peritoneal model, which consequently caused a proliferation decline and caused cell death [30]. Another study has targeted PDAC using oral MSCs [31]. The above discussed studies suggested MSCs as a promising therapy for targeting PDAC and other pancreatic cancer. A generally activated pathway believed to be involved in PDAC pathogenesis is the WNT signaling pathway. The WNT pathway has been controlled by MSCs through the upregulation of dickkop-related protein 1 expression that further disturbs the cell cycle in tumors [32]. In prostate and colon tumor cells, MSCs have also been shown to promote fibroblast cell proliferation and angiogenesis. Therefore, when interacting with tumor cells, MSCs tend to act as a double edge sword [33].

### 3.1. Cancer Stem Cells 

**Figure 2:**
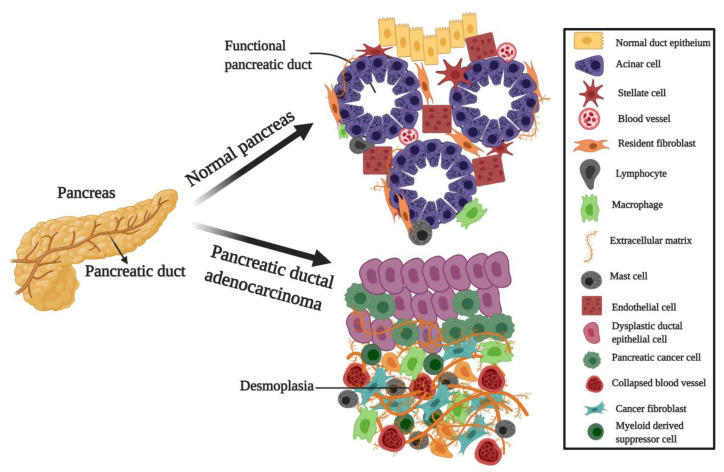
Pancreatic ductal adenocarcinoma microenvironment. Normal/healthy pancreas: pancreatic ducts and acinar cells are lined by epithelial cells in the healthy exocrine pancreas, the peri-acinar space is surrounded by resident fibroblasts and pancreatic stellate cells, and the extracellular matrix is mainly confined to interlobular septa and pancreatic ducts. Pancreatic ductal adenocarcinoma (PDAC) cells invade the basal membrane of dysplastic pancreatic ducts and infiltrate the healthy tissue. This infiltration is followed by a heavy desmoplasia in which pancreatic stellate cells are activated to tumor fibroblasts, and extracellular matrix components are synthesized in excess—figure created with BioRender.com and modified from [12].

Cancer stem cells (CSCs) are mainly responsible for metastasis, recurrence of tumors, and resistance to anti-cancer drugs in pancreatic cancer, including PDAC [34]. The first existence of CSCs was reported in 1997 [35]. Proper understanding of pancreatic cancer stem cells (PCSCs) is a promising way to develop new opportunities for prevention and prognosis; PCSCs prompted new therapeutic methods to be explored that can specifically target cancer cells. CSCs are an unusual cancer cell population capable of self-renewal and division [36]. Cancer cells are found in a special niche comprised of a hypoxic/necrotic microenvironment comprising fibroblasts, perivascular cells, endothelial, and immune, along with extracellular matrix elements, growth factors, and cytokines (Figure 2). The plasticity of CSCs is another important factor that plays a major role in tumor progression and therapeutic resistance, as they have an increased ability to adapt to challenges or reprogram their metabolism and presented by drug therapy and the tumor microenvironment [36,37]. CSCs shift their microenvironment by attaining intermediate metabolic phenotypes and shifts their metabolism from oxidative phosphorylation to glycolysis. The main factors and signaling pathways which are necessary for self-renewal, resistance to available strategies, and epithelial to mesenchymal transition (EMT) consist of WNT/β-catenin, Notch, and Sonic Hedgehog (SHH). PCSCs are highly resistant and can withstand traditional therapies that interfere with the complete eradication of cancerous cells [38]. Interestingly, PCSCs co-exist with other components of the tumor microenvironment, and to improve the understanding of PCSCs biology, it is essential to understand the correlation between these factors and PCSCs [34]. Chemo-resistance, together with metastatic potential, are the main clinical hallmarks of PCSCs. Metabolic inactivation and efflux of the drug from the cells as well as dysregulation/mutations in the drug targets, are responsible for the chemo-resistance of CSCs [38,39].

### 3.2. Potential Approaches Targeting PCSCs

Formulation of anti-cancer drugs to target associated proteins and pathways, thus improving chemotherapeutic efficacy [40]. Destruction of PCSCs should be able to avoid further tumor growth [41]. Prospective approaches are discussed below to target PCSCs. Anti-cancerous and non-cancer related drugs are listed in Table 1, whereas some drugs are used in combination (Table 2).

## 4. Signaling Pathways Involved in PCSCs

Different signaling pathways: PI3K/AKT [67,68], JNK [69], MEK/ERK [70], nuclear factor kappaB (NF-κB) [71], TGF-β [72], WNT [73,74], and Hedgehog [75,76] are known to crosstalk with Notch signaling pathway or with each other in the progression of pancreatic CSCs [77,78,79], because of their crucial role in production and differentiation of pancreatic cells [80,81]. It is therefore believed that the interaction between these signaling pathways and Notch signaling pathway can play an important role in the development of pancreatic tumors [78]. In CSCs and the EMT process of pancreatic cancer, signaling pathways are altered and are involved in CSC self-renewal, tumor formation, metastasis, invasion, and resistance to chemo-radiation [82]. There are various studies in which researchers have investigated new treatment strategies to control these pathways in order to cure cancer [77,83].

### 4.1. Notch Signaling Pathway

Notch signaling is one of the conserved signaling pathways accountable for cell-to-cell direct contact. The pathway of notch signaling is associated with pancreatic cell′s survival, proliferation, apoptosis, development, differentiation, and can promote EMT. It is essential for various aspects of cancer biology: angiogenesis, CSCs, and tumor immunity [84]. Notch signaling pathways have been shown to promote vascular endothelial growth factor (VEGF) and cellular migration in pancreatic cancer cells by activating NF-κB [85]. The Notch pathways consist of five canonical ligands (Delta-like ligand 1 (DLL1), DLL3 and DLL4, and Jagged1 and 2) and four single-pass transmembrane receptors (Notch1 to Notch4) [86,87]. Different ligands and receptors of Notch are expressed by various types of tumor. Notch signaling causes pancreatic Nestin^+^ precursor cells accumulation and extend the ductal epithelium. Notch is associated with pancreatic cancer cell development by retaining epithelial cells in a progenitor state. Tumor cells exhibit Notch signaling over-expression, high Notch-1, and Notch-2 levels, while a poor expression of Notch signaling related factors is exhibited by the normal pancreas. Midkine is a heparin binding growth factor, interacts with Notch2 in chemo-resistant PDAC, leads to increased expression of EMT and Notch pathway markers [88]. Studies of prostate cancer have shown increased marker expression of EMT and Notch1, including vimentin, N-cadherin, ZEB1 (Zinc-finger E-box binding homeobox), NF-κB, and PDGF-D in tumor cells [89]. In particular, two members of the Notch signaling pathway are involved in pancreatic cell development and carcinogenesis [90]. Few studies have shown increased Notch2 expression in PDAC rather than Notch1; Notch1 is activated during early pancreatic development, while Notch2 is mainly concerned with the branching of the ductal epithelium [91]. Furthermore, researchers have identified Notch1 and Notch2 as the main determinant in pancreatic carcinogenesis [92,93]. Overexpression of Notch2 and Jagged1 has been shown in gemcitabine resistant pancreatic cancer cells, whereas Notch1 is a crucial downstream mediator of Kirsten rat sarcoma viral oncogene homology (KRAS), and control tumor sphere formation of pancreatic cells [94,95]. In pancreatic cancer, the oncogenic KRAS mutation is the main factor, provides irreversible protein KRAS induction, and functions as a signaling molecule to trigger different transcription factors and intracellular signaling pathways related to cell proliferation, differentiation, migration, and survival [96]. High expression of Notch-1 and Jagged-1 has been associated with poor prognosis and patient survival in primary breast cancers [97]. DLL4 ligand overexpression in pancreatic cancer cells stimulates OCT4 and NANOG (pluripotency markers) expression, thereby increasing the number of CSCs [98]. Notch pathway activation is mainly responsible for the resistance of PCSCs to chemotherapy, yet the precise mechanism behind this remains poorly understood [95,99]. However, several pieces of evidence have shown that the Notch signaling pathway plays an important role in the development of pancreatic cancer, like supporting KRAS, and for the transformation of normal cells to tumor stem cells [90]. The Notch signaling pathway is triggered by several enzyme complexes, including γ-secretase complexes following Notch-ligand interaction and three consecutive proteolytic cleavages [84]. Notch1 inhibition induces increased the rate of apoptosis, migration, and intrusive properties of pancreatic cancer cells with γ-secretase inhibitors [100]. In 2018, Song et al. performed an experiment to evaluate the expression and possible therapeutic importance of Notch ligands and receptors in human PDAC [101]. The increased expression of Notch1 and 3 in PDAC tissues was observed, suggesting that both of these receptors may play an important role in the development of pancreatic cancer, and researchers have therefore referred to these receptors as oncogenes. On the other hand, the level of Notch 2 and 4 receptor and Notch ligands (DLL-1, 3, and 4) has been found to be decreased and increased, respectively [101]. Furthermore, Notch signaling pathway inhibition in the treatment of pancreatic cancer can be very attractive since there is no evidence disagreeing that Notch signaling plays a crucial role in pancreatic cell development, and addressing Notch as a remedy for pancreatic cancer might inhibit CSCs resistance to chemotherapy [102,103]. 

### 4.2. Hedgehog Signaling Pathway

The hedgehog signaling pathway is associated with the development, proliferation, and differentiation of embryonic cells [104] and also regulates healthy and malignant stem cells [105]. Researchers have shown that the Hedgehog pathway normally dormant in adult organs while remains active in cancer cells, where extracellular matrix production, myofibroblast differentiation, and stromal hyperplasia can be increased, allowing the EMT process in cancer cells [75,106,107]. The activation of the Canonical Hedgehog signaling pathway is represented by the Hh ligands′ (Sonic (SHH), Indian (IHH), and Desert (DHH)) interaction with the receptor Patched1 [108,109]. In 70% of pancreatic cancer tissue, overexpression of SHH suggests that Hedgehog signaling may be responsible for pancreatic cancer [110]. PANC-1 (pancreatic cancer cell line) studies have shown that smoothened suppression inhibits Hedgehog signaling, which can reverse EMT and induces apoptosis by inhibition of PI3K/AKT, and prevents pancreatic cancer cell invasion [111]. The hedgehog signaling pathway is a crucial objective for the development of chemotherapy [112]. The progression of many forms of cancer, including pancreatic cancer, is characterized by abnormal Hedgehog signaling pathway activity [113]. The survival of CSCs has been impaired by targeting Notch and Hedgehog signaling together, which indicates that these two pathways should be targeted at once in order to successfully eradicate certain forms of cancer [114]. Schreck et al. found that Notch suppresses Hedgehog directly by Gli1 transcription inhibition mediated by Hes1 [115]. Gli1 and Gli2 components of the Hedgehog are positively capable of controlling Hes1 regardless of the Notch pathway [116]. Concomitantly targeting both pathways could be more efficient in curing cancer. Notch pathway′s downregulation contributes to the inhibition of pancreatic cell growth and apoptosis, while the inhibition of Hedgehog leads to advancement in drug delivery to tumors [117]. The cancer stem cell maintenance, tumor-stroma cross-talk, and chemo-resistance could also be affected by interactions between Hedgehog and other signaling pathways. Mohelnikova et al. did not found any strong correlation between Hedgehog expression profile and KRAS mutation status in PDAC patients [118], regardless of previous PDAC studies [119]. Taxoid involvement with a dysregulated Hedgehog signaling pathway in patients with PDAC could have significant therapeutic benefits. New-generation taxoids can abrogate the overexpression of Hedgehog in pancreatic cancer [118]. Molecular interaction between WNT/β-catenin, Notch, TGF-β, and hedgehog indicates that during oncogenesis, two or more pathways crosstalk with each other. Thus in order to target different signaling pathways at once, a combination of novel inhibitors and traditional anti-tumor therapy are required to increase the effectiveness.

### 4.3. WNT Signaling Pathway

A significant embryonic signaling pathway for the morphogenesis, proliferation, and differentiation of various tissues, including the pancreas, is the WNT signaling pathway [120]. The WNT signaling consists of three main pathways: the canonical WNT pathway, non-canonical planar-cell polarity, and the non-canonical WNT calcium pathway [121]. Essentially, suppression of WNT signaling, along with Notch and Hedgehog signaling pathways, has led to the expansion of agents capable of inhibiting tumor invasiveness, metastasis, and carcinogenesis. In several forms of cancers, the WNT/β-catenin pathway regulates the self-renewal, proliferation, apoptosis, migration, and differentiation of stem cells [122]. Chemo-resistance in pancreatic cancer is also correlated with the impairment of the WNT/β-catenin pathway [122]. Liu et al. suggested that initiated β-catenin may increase self-renewal capability, reduces differentiation rate, and develop epithelial cancers [123]. A common characteristic of several kinds of pancreatic cancer is the activation of WNT signaling. In unusual tumor forms: acinar cell carcinoma, strong pseudo-papillary neoplasm, and pancreatoblastoma; mutation in canonical WNT/β-catenin components are generally observed [124]. WNT signaling is frequently triggered in PDAC, regardless of the lack of the normal phenomenon of mutations in pathway components. Sano et al. demonstrated that the activation of non-canonical signaling pathways contributes to the development of tumors [125]. *CYR61* expression is triggered by activation of the WNT/β-catenin pathway, and *CYR61* in response stimulates WNT/β-catenin signaling (feedback mechanism). Thus, disruption of this process represents a potential opportunity for therapeutic treatment [125]. WNT signaling influences many aspects of pancreatic biology, and its activity are constantly increased during pancreatic carcinogenesis. In pancreatic cancer, activated WNT target genes and accumulation of β-catenin have been observed [126]. Both Notch and WNT signaling pathways are important pathways that control stem cell differentiation and proliferation [127]. The Notch signaling pathway encourages pancreatic lineage commitment and pancreatic progenitor cell differentiation, whereas WNT signaling controls the stem cell state [128,129]. The Notch pathway is regulated by WNT signaling through the negative influence of WNT on the activity of GSK3-β mediated by *Dishevelled-2*. WNT and Notch signaling pathways seem to be interconnected; the Notch pathway functions as a negative controller β-catenin dependent pathway both in pancreas development and oncogenesis [73].

## 5. Epithelial to Mesenchymal Transition

The EMT seems to be a major factor in various natural biological processes, such as wound healing, embryogenesis, and cancer development. Evidence suggests that abnormal initiation of the EMT and developmental program leads to tumor initiation, metastasis, invasion, and therapeutic resistance [94,130]. EMT is a greatly harmonized process noticed when more or less epithelial features are lost by epithelial cells and embark on attaining mesenchymal cell characteristics, which is a crucial step during embryogenesis [131]. By stimulating multiple EMT transcription factors such as Twist, ZEB, Snail, and Slug; TGF-β, WNT, Notch, and Hedgehog signaling facilitated the conversion of epithelial cells into mesenchymal cells. In several cancer types, specific EMT signaling pathways such as Notch have been observed to be dysregulated, and activation of these signaling is often associated with poor clinical outcomes [132]. Dysregulation of TGF-β and Notch pathways of EMT plays an essential role in cancer and cardiovascular diseases [133]. WNT pathway in EMT regulates the levels of GSK3-β, β-catenin, Snail, and other processes linked with tumor progression [134]. It has been shown that EMT plays a key role in cancer cell resistance to traditional chemotherapeutics, including gemcitabine, vincristine, oxaliplatin, and taxol [135]. Gemcitabine resistant PCSCs showed mesenchymal morphology with activation of Alpha-SMA, Nestin, vimentin, and fibronectin and downregulation of β-catenin and E-cadherin [132]. The above-discussed studies have proven that dysregulation of different signaling pathways involved in EMT is crucial in tumor progression.

## 6. Role of Epigenetics

The complex interaction between genomic, epigenomic, and signaling pathway alterations affect PDAC growth and progression [136]. It has been shown that the EMT leads to the malignant phenotype in PCSCs. Extensive research regarding genetics and patterns of genome-wide expression suggests that genetic alterations are important for the initiation and early development of PDAC. Epigenomic studies, however, have shown that epigenetic changes in cancer cells and tumor suppressor genes have affected tumor growth [137]. Epigenetic modifications are legacy modifications of DNA or chromatin structures that affect gene expression without altering the sequence of DNA [138]. Antagonism between stemness inhibiting micro-RNAs and *ZEB1* has been shown to contribute to the EMT process for PCSCs [139]. Schmalhofer et al. have shown that *ZEB1* promotes the EMT process by controlling the related protein binding domains such as the p300-P/CAF binding domain, Smad interaction domain, and C-terminal-binding protein interaction domain [140]. Moreover, *ZEB1* stimulates EMT by blocking *E-cadherin* [141]. Moreover, Zhang et al. demonstrated the link between *ZEB1* and epigenetic regulation of EMT [142]. The *ZEB1* promotes the epigenetic silencing of *E-cadherin* through the incorporation of various *E-cadherin* promoter chromatin enzymes such as histone deacetylases, DNA methyltransferase, and ubiquitin ligase [142]. The drug resistance of PCSCs has also been shown to be associated with *ZEB1* [143]. *ZEB1* plays a crucial role in EMT during tumor carcinogenesis [144]. However, the clinical efficacy of *ZEB1* for solid human tumors remains uncertain. In order to determine the prognostic importance of *ZEB1* in patients with solid tumors, Chen et al. conducted a meta-analysis to determine the prognostic importance of *ZEB1* in patients with solid tumors [144]. The elevated *ZEB1* expression indicates poor survival in solid tumor patients. This study suggested that *ZEB1* could be a possible biomarker and potential therapeutic target for prognosis in solid human tumors [144]. 

## 7. Role of G Protein-Coupled Receptor in Pancreatic Cancer

The prospective of G protein-coupled receptors (GPCRs) in pancreatic adenocarcinoma and GPCR-targeted drugs as potential therapeutics for pancreatic cancer have been outlined in this section. The largest family of plasma membrane cell surface receptors is GPCRs (seven trans-membrane domain receptors), with >800 human members: ~400 are endoGPCRs that, in contrast to chemosensory GPCRs, respond to endogenous agonists (e.g., hormones and neurotransmitters). Being a family of cell surface receptors, EndoGPCRs are the largest family with targets for approved drugs, providing access from the extracellular environment. Signaling from GPCR affects different aspects of cancer like invasion, remolding, migration, etc. Functional aspects of GPCR have been established in cancers, including pancreatic cancer, both in the cells of cancer and tumor microenvironment. A wide variety of GPCRs are expressed by pancreatic adenocarcinoma tumor cells [145]. As the GPCRs are the largest drug target family, about 34% of all drugs approved by the Food and Drug Administration (FDA) targeted 108 members of GPCR [146]. In 2018, the worldwide sales of these drugs cost approximately 180 billion US dollars [146]. For the treatment of various diseases, GPCR targeted drugs have been used. GPCRs have been assessed as targets for about 50% of drugs available on the market due to their primary involvement in signaling pathways linked to many diseases, i.e., metabolic, immunological, mental, cardiovascular, sensory, inflammatory, and cancers. As GPCR play an important role in controlling signaling pathways involved in cancer, they are used as biomarkers for the early prognosis, and only a few receptors are represented by GPCR [147]. This is primarily due to drug resistance; receptor desensitization has been observed in experiments utilizing long and short-term exposure to GPCR-targeting drugs [147,148]. In order to develop new GPCR based therapy, more research is required on the downstream regulators and pharmaceutical potential of GPCRs, which can efficiently control cancer cell pathways. Additionally, most cancer forms, including PDAC, female sex is related to poor prevalence and better therapeutic results. The underlying mechanism behind this sex-based incidence was unclear. Regardless of the fact that PDAC lacks basic nuclear estrogen receptors, Natale et al. believed that estrogen signaling could be responsible for sex-based incidence [149]. They have used synthetic agonist G-1 (small molecule) that activates G protein-coupled estrogen receptor (GPER), which have been used to inhibit PDAC by GPER estrogen receptors present on tumor cells. Subsequently, it contributed to PDAC proliferation inhibition, reduced PDL-1 (programmed death ligand-1), c-Myc, and increased immunogenicity of the cells [149]. Researchers found that the G-1 contributes to inhibition of melanoma, suggesting that GPER agonists could be helpful against a large range of cancers (cancers of the lung, adrenal gland, bone, colon, and skin etc.) with the exception of sex hormone related cancers [149,150].

## 8. Role of Cysteine in Pancreatic Cancer

Another approach for PDAC is to target essential biological processes involved, especially in PDAC cells. Depletion of cysteine causes ferroptosis (programmed cell death dependent on iron and characterized by the accumulation of lipid peroxides) of pancreatic cancer cells [151]. Ferroptosis is a type of programmed cell death induced by the disastrous production of reactive oxygen species (ROS). In several tumor types, lipid ROS production is increased by oncogenic signaling pathways and is counterbalanced by amino acid cysteine derived metabolites; exogenous cysteine is imported by cystine/glutamate transporter (Xc- system). The Xc- system inhibition has been shown to promote ferroptosis in many cancer cell lines; therefore, in pancreatic cancer, cysteine is required to inhibit ferroptosis [152]. PDAC cells circumvent the consequences of increased ROS production, which can be caused by mutational activation of KRAS, as one example by upregulating metabolic processes that yield cysteine-derived metabolites, such as glutathione, that reduce ROS levels. Hypothesizing that cysteine import would be a key to the survival of PDAC cells, Badgley et al. performed in vitro experiments using human PDAC cell lines, which confirmed that PDAC cells depend on cysteine import. Specifically, PDAC cells deprived of cysteine by various means (including inhibition of the cysteine importer system Xc- underwent ferroptosis [153]. In the PDAC mouse model, deletion of the gene encoding a subunit of system Xc- (Slc7a11) in established tumors substantially increased median survival and even caused complete tumor regression in one mouse. Tumors in which Slc7a11 was deleted exhibited microscopic characteristics abnormal for PDAC tumors, including large lipid droplets and structurally defective mitochondria, and had increased expression of ferroptosis-related genes. Interestingly, although previous work found that cysteine′s role in ferroptosis was related to the synthesis of glutathione, inhibition of glutathione biosynthesis alone in human PDAC cell lines did not increase lipid ROS levels or cause ferroptosis. Instead, a reduction in levels of coenzyme A, also synthesized from cysteine, cooperated with glutathione loss to induce ferroptosis [153]. Notably, in a mouse model of PDAC, treatment with cystinase, which degrades cysteine, caused tumor stabilization or regression in all mice. Tumors from these mice exhibited the characteristic signs of ferroptosis observed with the Xc^–^ system (Slc7a11) deletion, including abnormally large lipid droplets and mitochondrial aberrations. Collectively, these results provide further evidence that PDAC is dependent on cysteine metabolism to prevent ROS-induced ferroptosis and suggest that cysteine depletion may be a useful clinical strategy [153]. As cysteine import is related to the pancreatic cancer cell′s survival, researchers proposed that cancer growth can be inhibited by selectively targeting cysteine. Researchers observed that cystine starvation leads to the reduction of glutathione in cells, and vitamin E can be used to revive cysteine starvation-induced cell death, thus suggesting the role of induced oxidative cell death in this case [154]. Cysteine is required for glutathione production inside the cancer cell, which it uses as a defense [153]. Chemotherapy used without sulfasalazine (Xc^–^ system inhibitor) is ineffective in PDAC. Sulfasalazine, an old cheap off-patent drug, is used alongside artesunate and intravenous vitamin C, which at a high dose, produces hydrogen peroxide free radicals, a cocktail that is also combined with iron to effectively induce ferroptosis. Researchers have demonstrated that cysteinase, a new drug compound, can starve pancreatic cells of cysteine supply, causing ferroptosis [153].

## 9. Role of Pyruvate Kinase M2 

Under aerobic and anaerobic environments, differentiated cells prefer oxidative phosphorylation and anaerobic glycolysis, respectively [155]. In comparison, even in the presence of oxygen (the Warburg effect), proliferative tissue and cancer cells appear to intake a large quantity of glucose to generate lactate through glycolysis. The Warburg effect is mediated by the pyruvate kinase M2 (PKM2) isoform [156]. Conversion of phospho-enol-pyruvate and adenosine diphosphate into pyruvate and adenosine triphosphate is catalyzed by pyruvate kinases. There are four isoforms of Pyruvate kinase (L, R, M1, and M2: PKL is present in the liver, PKR is expressed by red blood cells, PKM1 present in most differentiated tissues, and PKM2 expressed in embryonic and cancer cells [156,157]. PKM2 is highly expressed in different forms of human cancer, including pancreatic cancer [158,159]. As earlier discussed, PDAC cells change their metabolism from mitochondrial to glycolysis, which fuels the plasma membrane calcium pump (PMCA), ultimately prevents Ca^2+^ induced cell death; PDAC has low survival and few possible treatments [160].

In PDAC cells, glycolytic ATP production inhibition promotes cytotoxic Ca^2+^ accumulation and cell death, as researchers showed that increased glycolytic rate is important for fueling the ATP dependent PMCA [161], PMCA′s dependency on glycolytic ATP seems to be a possible therapeutic option. Identifying the molecular mechanism behind the dependency of PMCA on glycolytic ATP could reveal novel therapeutic goals for the development of effective drugs. PKM2 is a major glycolytic enzyme-producing oncogenic ATP, especially over-expressed in pancreatic cancer [162,163]. Furthermore, effective and selective inhibitor of PKM2 is shikonin, anti-inflammatory, antimicrobial, and anti-cancer effects of shikonin have been reported [164]. In PDAC, shikonin is a valuable method for investigating the supply of PKM2-mediated ATP to PMCA. For cell survival, a functional relationship is important between PMCA and PKM2. Shikonin reduces cell viability, proliferation, PDAC migration and induces cell death [160]. Researchers demonstrated that elevated expression of PKM2 is related to poor recovery in human PDAC. The PKM2 downregulation leads to lower intracellular metabolites level and glycolytic activities [162]. Additionally, various cancer properties in pancreatic cancer cells were suppressed by shikonin. Recently, James et al. performed a study where they have cut off the supply of glycolytic ATP to the pancreatic cancer cells PMCA; it would be comparatively extra effective in cancer cells than non-cancerous cells [160]. In metabolic activities, as well as in PDAC cell malignancy, PKM2 plays a significant role.

## 10. Repurposed Drugs for CSCs 

In this section, we discussed some non-cancer targeted drugs with anti-cancer effects against CSCs that can be used to treat PDAC. These drugs function through various mechanisms of action, along with the inhibition of certain significant pathways involved in PCSCs. Antibiotics are among the molecules that display extremely complex biological behaviors by interacting with the EMT and WNT pathways in breast CSCs [42]. Salinomycin has been shown to inhibit tumor growth and metastatic spread of PDAC in a genetically modified mouse model [165]. Nigericin has been shown to inhibit EMT, increase the expression of E-cadherin, and induce cell cycle arrest of CSCs that contribute to the reduction in cancer cell invasion and metastasis [43,166]. Furthermore, azithromycin has been shown to increase the overall survival rate in cancer patients by preventing the development of tumors in PDAC and other cancers [44]. Moreover, certain anti-malarial agents such as chloroquine have been shown to have important effects on PCSCs by inhibiting the Hedgehog pathways and CXCR4 [45]. Aspirin has the potential to be an effective adjuvant cancer therapy and might be a promising candidate for eradicating PCSCs in PDAC [46]. The phase III trial acknowledged the significant effect of aspirin as an alternative therapy to avoid disease recurrence [46]. Metformin showed a significant potential for pancreatic cancer chemoprevention through decreased PCSC markers and inhibition of the mechanistic target of rapamycin (mTOR), extracellular signal-regulated kinases (ERK), phosphorylated extracellular signal-regulated kinases (pErk), and insulin-like growth factor 1 (IGF-1) in a PDAC mouse model [167]. Another non-cancer related drug, disulfiram, is able to target PCSCs in PDAC by inhibiting ERK, proteasome, and NF-κB signaling pathway when used alone or in combination with chemo-radiation [47]. Additionally, a phase II clinical study showed that the combination of disulfiram and chemotherapy improved survival in newly diagnosed lung cancer patients [168]. The above discussed studies showed that the repurposing of existing compounds to target PCSCs could also be a potential approach to overcome PDAC.

## 11. Immunotherapy for Pancreatic Cancer

Several targeted strategies, including new stromal modulation, immunotherapeutic approaches, and targeting main signaling pathway effectors, are in progress, along with the development of novel cytotoxic therapeutic strategies. The stroma encompasses approximately 90% of the tumor mass, which promotes the progression of fibrosis and immunosuppression [169]. In addition to facilitating tumor development, the PDAC stroma has been shown to attenuate the delivery of antitumor treatments, inactivation of cytotoxic CD8^+^ T cells, and increasing the number of immunosuppressive cells [170,171]. During the progression of the disease, the number of pancreatic stellate cells and PDAC specific cancer associated fibroblasts increase abundantly [172]. These activated stellate cells promote tumor growth by reducing the migration of CD8^+^ T cells to juxtatumoral stromal compartments [173]. Stellate cells also stimulate T cell anergy and apoptosis induced by galectin-1, resulting in evasion of immune surveillance by the cancer cells [173,174].

Furthermore, B lymphocytes contribute actively to PDAC fibrogenesis by activation and differentiation of cancer associated fibroblasts [175]. Minici et al. reviewed the immunological mechanisms that promote and inhibit the anti-tumor immunity of B cells. B cells can restrict tumor growth through phagocytosis by macrophages, facilitating tumor killing by NK cells, generating tumor-reactive antibodies, and the priming of CD4^+^ and CD8^+^ T cells [176]. B cells can facilitate tumor growth through the production of autoantibodies and tumor growth factors [176]. Further, targeting particular B cell subtypes can be beneficial for the treatment of cancer as the activities of Th1, and CD8^+^ cytolytic T cells can be directly and indirectly inhibited by regulatory B cells. 

Presently, many clinical trials are trying to evaluate the efficiency of immunotherapeutic approaches in PDAC, including cancer vaccination [177], immune checkpoint inhibitors [178], monoclonal antibodies, adoptive cell transfer [179], chemo-radiotherapy or other molecularly focused agents, and combinations with other immunotherapeutic agents or immune modulators, though none of these studies have demonstrated improvements in practice. Activating a patient′s T cells is the key basis of cancer immunotherapy in order to destroy tumor cells. Furthermore, important steps of immunotherapy are defined as follows: reduction in tumor-specific cells presenting antigen, T cell activation, T cells infiltration into tumors, cancer cell recognition by T cells, and cancer cell elimination [180]. Anti-CTLA-4 (Ipilimumab) and anti-PD-1/anti-PDL-1 (Nivolumab/Pembrolizumab) agents have shown promising results in the activation of T cells and offer an efficient tumor immunotherapy strategy [181]. Despite showing Powerful outcomes of some malignancies, most of them in phase I and II clinical studies have not shown any clinical effectiveness in PDAC [182]. The immunosuppressive activity of CTL-4 results in the reduction of T effector cell activation and elevation in the activity of T regulatory cells [183]. The programmed cell death protein 1 (PD-1) is present largely on T cells, tumor cells, and tumor infiltrating lymphocytes [6]. The binding of PD-1 (Programmed death-ligand, PDL-1/PDL-2) leads to a reduction in T cell proliferation and secretion of antitumor cytokines [6]. 

A varied range of clinical trials (Table 3) on pancreatic cancer based on cytotoxic chemotherapy, vaccine-associated checkpoint inhibitors, immune checkpoint monotherapy, dual checkpoint combination therapy, and using other inhibitory agents have been completed or are presently ongoing. These clinical trials followed several therapeutic techniques:

1. Monotherapy includes the administration of several PD-1(MEDI4736, MPDL3280A, and pembrolizumab,) and CTL-4 (tremelimumab and ipilimumab) inhibitors and inhibition of double checkpoints: either by a combination of the above mentioned inhibitors or with other agents, such as anti-CCR-5 (mogamulizumab) [184].
2. Combination of chemotherapeutic agents and immune checkpoint inhibitors: PD-1/CTL4 inhibitors leads to the activation of T cell that is efficient for immunotherapy. When PD-1/CTL4 inhibitors combined with commonly used chemotherapeutic agents such as Nab-paclitaxel, gemcitabine, carboplatin, and FOLFOX improved overall survival [47]. Remarkably, therapeutic procedures using a combination of immune checkpoint inhibitors with radiotherapy or chemotherapy have shown significant outcomes [185,186].
3. Vaccination therapy is founded on the basis of the distribution of tumor antigens to antigen presenting cells (APCs), followed by induction of an organized immune response. Cancer specific DNA mutations produce new antigens, which, in turn, results in a unique sequence of the peptide. Variety of vaccines for pancreatic cancer treatment includes whole-cell vaccines, dendritic-cell based vaccines, peptide and DNA vaccines, telomerase peptide vaccines, Ras peptide vaccines, and survivin-targeted vaccines [187]; however, regardless of the enhanced immune system, showed poor clinical results. GVAX is an allogeneic irradiated whole-cell tumor vaccine genetically modified for the secretion of granulocyte macrophage colony stimulating factor and promotes cytolytic action against tumors, the most widely studied vaccine for pancreatic cancer [188]. Furthermore, the clinical studies when GVAX is applied in combination with 5-Fluorouracil/cyclophosphamide based chemotherapy have shown the same results regarding disease-free and median survival as that of GVAX applied alone [189]. On the other hand, when the above mentioned ipilimumab (immune checkpoint inhibitor) is applied in combination with GVAX, it leads to better survival [190].
4. Adoptive T cell immunotherapy is based on the modification of autologous T cells, which stimulates the immune response against the tumor. The patients receiving mesothelin-targeting chimeric antigen receptor-T (CAR) cells have shown overexpression of a membrane antigen in pancreatic cancer, exhibited adequate patience but unsuccessful in showing good results [191]. Along with mesothelin, other cancer-associated antigens are being studied alone or in combination with chemotherapy as potential targets of CAR-T cells based therapy [191].
5. Immune modulating agents that target the microenvironment of the pancreas can also exert extensive antitumor activity. Anti-CD40 agonistic antibodies used in combination with gemcitabine in PDAC patients showed significant results [192]. PDAC patients treated with a CCR2 inhibitor (PF-04136309) exhibited fractional response and constant tumor when used in combination with FOLFIRINOX [193]. Several chemokine receptor molecules are under examination in clinical trials against PDAC.

## 12. Natural Killer Cell Therapy 

The primary immune cells that attack cancer cells and can be used as a therapeutic agent against pancreatic cancer are natural killer (NK) cells. NK cells are a heterogeneous subgroup of immune cells that express a wide variety of activators and inhibitors; hence they are able to attack and destroy the tumor cells directly without the necessity for major histocompatibility complex (MHC) specificity. NK cells have developed to counterpart B cells and T cells in host defense against carcinogens and other pathogens. If any carcinogenic mutation takes place, NK cells quickly start destroying neighboring cells. This feature is unique among immune cells, and their tendency to enhance antibody production, T cell, and B cell proliferation means NK cells likely play a significant role as anti-cancer therapy [194]. While tumors can evolve various mechanisms to counterattack endogenous NK cell attacks, in vitro or ex vivo activation, expansion and, genetic modification of NK cells can intensify their anti-tumor activity and allow resistance to be overwhelmed. Through specific mechanisms, which depend upon various sets of inhibitory and stimulatory receptors, are responsible for the tumor cell recognition via NK cells [194]. Some studies suggested the importance of NK cells in PDAC and their potential therapeutic effect [195]. PDAC significantly impair functions of NK cell by downregulating the ability of cytokines secretion and effector molecules. The tumor microenvironment also plays a key role in the reduction of NK cells cytotoxicity and stimulates immune suppression by different pathways. An antibody based NK cell homing protein called NK cell recruiting protein conjugated antibody (NRP-body) developed by researchers improved the efficiency of NK-cell based therapy in the treatment of PDAC [196]. The effect of NRP-body on the penetration of NK cells into primary and metastatic pancreatic cancer has been evaluated in PDAC murine model. NK-cell infiltration induced by CXCL16 via RhoA activation through the ERK signaling. NRP-body administration to pancreatic cancer mice model augmented the penetration of relocated NK cells into tumor cells, and the tumor load was decreased than that of control. NRP-body treated groups showed overall increased survival than that of other groups treated with NK cells alone [196]. Increased diffusion of NK cells into tumor tissues strengthened the response. Thus, for the treatment of pancreatic cancer, the combination of NRP-body with NK cell therapy is more beneficial than NK cells used alone.

## 13. Discussion

We have tried to deliberate nearly all of the most recent updates about several methods to treat pancreatic cancer, mainly PDAC. Pancreatic cancer, metastasis of tumor at the early stages and the dearth of efficacious treatment is the main reason behind the poor survival and the increased cancer death rate. Patients with pancreatic cancer have severe immune deregulation, marked by the proliferation of immunosuppressive cells and increased pro-inflammatory cytokines [197]. Various researchers have demonstrated that the EMT process and CSCs are mainly responsible for chemo-resistance and the metastatic potential of tumor cells [39,198,199]. CSCs development is based on genetic mutations in the signaling pathways, which leads to the transformation of undifferentiated and differentiated stem cells [200]. Due to the destructive tumor malignancy of PDAC, effective therapeutic approaches are still required, especially in order to disrupt its tumor microenvironment or EMT or to minimize its resistance to therapeutic agents. In the presence or absence of apparent metastatic disease, targeting molecules that directly facilitate infiltration and metastasis should be considered crucial in the treatment of PDAC and other pancreatic cancer [85,201]. Additionally, immunotherapy can be improved via NK cells as a result of their increased invasion of pancreatic cancer cells [196]. Conventional therapy in combination with CSC inhibitors may offer an effective approach for the treatment of pancreatic cancer [39]. Although the actual mechanism of action behind targeting PCSCs is poorly understood, stem cells, especially MSCs, can be an ideal candidate for the treatment of PDAC and pancreatic disorders due to their homing ability, anti-inflammatory, and immunomodulatory properties [26]. Moreover, PDAC patients treated with hematopoietic stem cells after pancreaticoduodenectomy (Whipple procedure) have shown no signs of disease recurrence (https://clinicaltrials.gov/ct2/show/NCT02207985). MSCs offer a safer choice to cure PDAC as they do not exhibit immune rejection and teratoma formation [26]. Above mentioned studies suggest that MSCs transplantation will reduce the drug burden, inhibitor-associated side effects of pharmacotherapy and may prove to be useful for new drug testing.

## 14. Conclusions

PDAC remains a destructive disease with a poor survival rate and prognosis. This is due to the early stage tumor metastasis and the lack of any successful therapy. In the present review, we have summarized the current therapeutic strategies and the role of different factors like PCSCs, signaling pathways, and immunotherapy in PDAC. Recent advances in targeting PCSCs using effective drugs alone or in combination with MSCs have shown great potential in the treatment of PDAC by preventing CSCs development and proliferation. Different signaling pathways involved in PDAC are responsible for drug resistance that advocate the requirement of novel drugs to target these signaling pathways. Furthermore, sequencing and proteomics analysis is needed to identify the specific protein targets of signaling pathways involved in PCSCs to eliminate resistance against chemotherapy. Finally, an improved understanding of PCSCs related signaling pathways, identification of specific protein targets, and discovery of non-resistant drugs will guide successful PDAC therapies.

## Figures and Tables

**Table 1:** List of Food and Drug Administration (FDA) approved drugs and their potential effects on pancreatic cancer stem cells.

Drug		Structure		Pathway inVolved		Mechanism of Action		Accession Number		References
Salinomycin		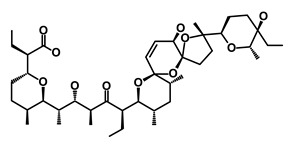		WNT/EMT		Inhibits the growth of CSCs		DB11544		[42]
Nigericin		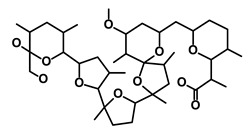		EMT		Inhibit the cell viability		DB14056		[43]
Azithromycin		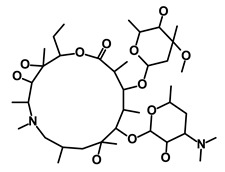		Mitochondria		Inhibiting protein synthesis and translation		DB00207		[44]
Chloroquine		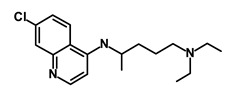		OXPHOS		Inhibits the autophagy pathway		DB00608		[45]
Aspirin		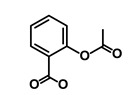		ALDH1, NF-κB		Blocks prostaglandin synthesis		DB00945		[46]
Disulfiram		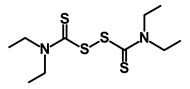		WNT, β-catenin and NF-κB		Induce apoptosis in cancer stem cells		DB00822		[47]
Aprepitant		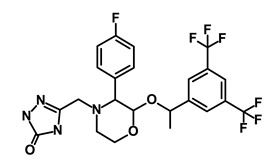		WNT		Inhibit emesis induced by cytotoxic chemotherapeutic agents		DB00673		[48]
Atovaquone		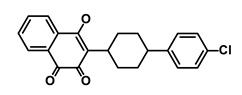		HER2/β-catenin		Antiprotozoal, antimicrobial and antipneumocystis activity		DB01117		[49]
AZD8055		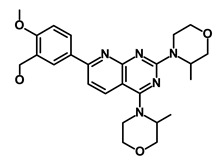		mTOR		Activation of epidermal growth factor receptor		DB12774		[50]
Crocetinic acid		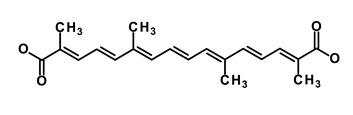		Hedgehog		Inhibited epidermal growth factor receptor and Akt phosphorylation		NA		[51]
GANT-61		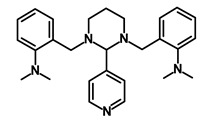		Hedgehog		Inhibited cell viability. protects autophagy		NA		[52]
Ketamine		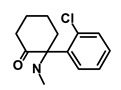		WNT		Inhibiting proliferation, invasion, and migration		DB01221		[53]
Metformin		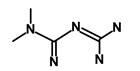		mTOR, PI3K/Akt		Antineoplastic activity		DB00331		[54]
Quinomycin A		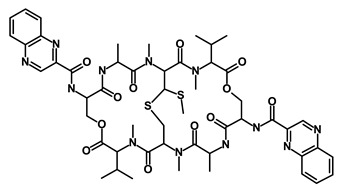		Notch		Suppresses CSCs growth		DB15582		[55]
Rapamycin		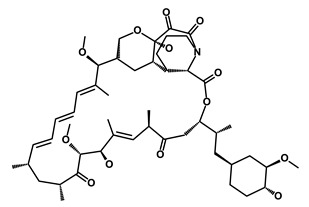		mTOR		Reduced the viability of CSCs		DB00877		[56]
RO-4929097		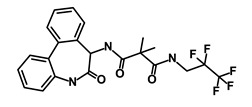		Notch		Suppresses the tumor initiating potential of cancer cells		DB11870		[57]
Sanguinarine		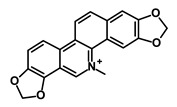		Hedgehog		Inhibits the growth of CSCs		NA		[58]
Tigecycline		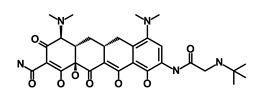		OXPHOS		Inhibits cell proliferation, migration and invasion		DB00560		[59]
5-FU (Fluorouracil Injection)		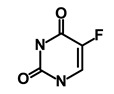		Antineoplastic antimetabolite		Inhibition of the formation of thymidylate from uracil		DB00544		[60]
Mitomycin		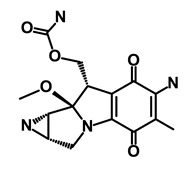		Antineoplastic antibiotic		Alkylating agent which inhibits DNA synthesis		DB00305		[60]
Abraxane/Paclitaxel		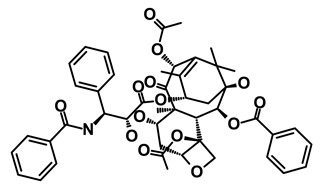		Microtubule associated protein		Stabilizes microtubules by preventing depolymerization		DB01229		[61]
Gemcitabine Hydrochloride/Gemzar		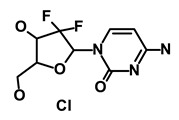		Antineoplastic anti-metabolite		Inhibits thymidylate synthetase		DB00441		[61]
Afinitor/Everolimus		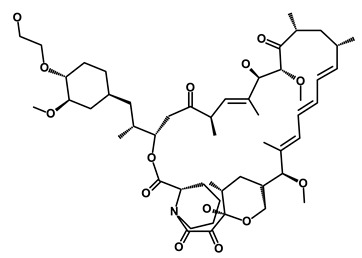		mTOR inhibition		Immunosuppressant		DB01590		[62]
Erlotinib Hydrochloride/Tarceva		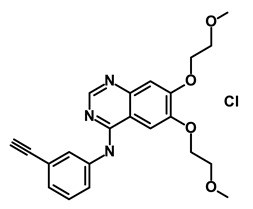		Epidermal growth factor receptor (EGFR)		Inhibits the intracellular phosphorylation of tyrosine kinase		DB00530		[63]
Lynparza/Olaparib		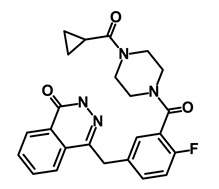		Poly (ADP-ribose) polymerase (PARP) inhibitor		Inhibit growth of tumor cells		DB09074		[64]
Onivyde/Irinotecan		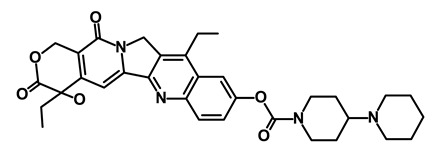		Antineoplastic enzyme inhibitor		Inhibits the action of topoisomerase I		DB00762		[65]
Sunitinib Malate/Sutent		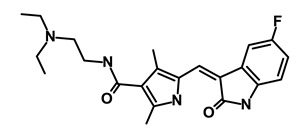		Multi-targeted receptor tyrosine kinase (RTK) inhibitor		Inhibits cellular signaling by targeting multiple RTKs		DB01268		[66]

**Table 2:** Drug combinations used in pancreatic cancer.

Drug	Accession Number	Function	Mechanism of Action
FOLFIRINOX			
FOL: Folinic acid/Leucovorin	DB00650	Antidote	Enhances the effects of 5-fluorouracil
F: Fluorouracil	DB00544	Pyrimidine analog and antimetabolite	Inhibit DNA synthesis
IRIN: Irinotecan/Camptosar	DB00762	Topoisomerase inhibitor	Prevents DNA from uncoiling and duplicating
OX: Oxaliplatin/Eloxatin	DB00526	Platinum-based antineoplastic agent	Inhibits DNA repair and synthesis
GEMCITABINE-OXALIPLATIN			
Gemcitabine	DB00441	Antineoplastic anti-metabolite	Inhibits thymidylate synthetase
Oxaliplatin	DB00526	Platinum-based antineoplastic agent	Inhibits DNA repair and synthesis
GEMCITABINE-CISPLATIN			
Gemcitabine	DB00441	Antineoplastic anti-metabolite	Inhibits thymidylate Synthetase
Cisplatin	DB00515	Antineoplastic	Alkylating agents
OFF			
O: Oxaliplatin	DB00526	Platinum-based antineoplastic agent	Inhibits DNA repair and synthesis
F: Fluorouracil	DB00544	Antineoplastic antimetabolite	Inhibition of the formation of thymidylate from uracil
F: Folinic Acid/Leucovorin	DB00650	Antidote	Enhances the effects of 5-fluorouracil

**Table 3:** Clinical trials of novel agents for PDAC and other pancreatic cancers.

Pathological Condition	Enrolled Patients	Intervention	National Clinical Trial Number	Outcome Measures	Phase	Status	Result
Neoplasms, Pancreas	40	Cancer stem cell vaccine	NCT02074046	Determine the safety of immunization	Phase 1/2	Completed	CTLs harvested from CSC-vaccinated hosts were capable of killing CSCs in vitro
Metastatic pancreatic cancer	98	Gemcitabine, Nab-Paclitaxel, GDC-0449	NCT01088815	Progression free survival, safety of combination therapy	Phase 2	Completed	Median progression-free survival and overall survival were 5.42 months and 9.79 months, respectively
Metastatic pancreatic adenocarcinoma	139	BBI608 either in combination with Gemcitabine and nab-Paclitaxel, mFOLFIRINOX, FOLFIRI, or MM-398 with 5-FU and Leucovorin	NCT02231723	Safety, Adverse effects	Phase 1	Completed	Inhibit cancer stemness pathways, including Nanog, by targeting stemness kinases.
Metastatic Pancreatic Ductal Adenocarcinoma	65	MEDI4736 Monotherapy, Tremelimumab + MEDI4736	NCT02558894	Response Rate, Overall survival, progression free survival,	Phase 2	Completed	Monotherapy reflected a population of patients with mPDAC who had poor prognoses and rapidly progressing disease
PDAC, Pancreatic Cancer	21	Ipilimumab, Gemcitabine hydrochloride	NCT01473940	Overall survival, progression free survival, recovery of tumor immune surveillance	Phase 1	Completed	Median progression-free and overall survival were 2.78 months and 6.90 months, respectively.
Second-line, third-line and Greater Metastatic Pancreatic Cancer	303	GVAX Pancreas Vaccine, CRS-207, Chemotherapy, Cyclophosphamide	NCT02004262	Overall survival and adverse effects	Phase 2	Completed	Median overall survival in the primary cohort was 3.7, 5.4, and 4.6 months in arms A, B, and C, respectively (*)
Pancreatic Neoplasm	22	Monoclonal antibody, chemotherapy	NCT00711191	Overall survival, progression free survival, and time to Progression	Phase 1	Completed	Well tolerated and associated with antitumor activity in patients with PDAC and improved overall survival
Pancreatic Adenocarcinoma metastatic	10	Melphalan, BCNU, Vitamin B12, Vitamin C, and autologous hematopoietic stem cell	NCT04150042	Response rate in metastatic lesions, overall survival, progression free survival	Phase 1	Ongoing	NA
Resectable pancreatic adenocarcinoma	42	HIPEC-Gemcitabine	NCT03251365	Morbidity, survival	Phase 2/3	Ongoing	NA
PDAC, pancreatic cancer, metastasis	36	Ascorbic acid, Paclitaxel, Cisplatin, Gemcitabine	NCT03410030	Determination of preliminary efficacy	Phase 1/2	Ongoing	NA
Pancreatic Cancer	81	Pembrolizumab, Gemcitabine, Docetaxel, Nab-paclitaxel, Vinorelbine, Irinotecan, Liposomal Doxorubicin	NCT02331251	Determine the recommended phase 2 dose	Phase 1/2	Terminated	The median progression-free survival and overall survival was 9.1 and 15.0 months, respectively
Pancreatic Cancer	15	Fludarabine, Anti-mesothelin chimeric T cell receptor (CAR) transduced peripheral blood lymphocytes (PBL), Cyclophosphamide, Aldesleukin	NCT01583686	Tumor regression response and adverse effects	Phase 1/2	Terminated	MORAb-009 (chimeric monoclonal antibody) is well tolerated
Pancreatic adenocarcinoma	10	Allogeneic hematopoietic stem cell transplantation	NCT02207985	Disease free survival	Phase 1/2	Unknown	Patients are tumor-free for 9 years after diagnosis

* CTLs: cytotoxic T lymphocytes; Cy/GVAX + CRS-207 (arm A), CRS-207 (arm B), or physician′s choice of single-agent chemotherapy (arm C); HIPEC: Hyperthermic Intraperitoneal Chemotherapy; NA: Not available.

## Data Availability

Not applicable.

## Abbreviations

CSCs	Cancer stem cells
EMT	Epithelial mesenchymal transition
ESCs	Embryonic stem cells
GPCR	G protein-coupled receptor
GPER	G protein-coupled estrogen receptor
MSCs	Mesenchymal stem cells
NK cell	Natural killer cells
PCSCs	Pancreatic cancer stem cells
PDAC	Pancreatic ductal adenocarcinoma
PKM2	Pyruvate kinase M2
PMCA	Plasma membrane calcium pump
ROS	Reactive oxygen species
SHH	Sonic Hedgehog
Xc-	Cystine/glutamate transporter
ZEB	Zinc-finger E-box binding homeobox
